# Enablers of the Implementation of Tissue Plasminogen Activator in Acute Stroke Care: A Cross-Sectional Survey

**DOI:** 10.1371/journal.pone.0114778

**Published:** 2014-12-09

**Authors:** Alice Grady, Jamie Bryant, Mariko Carey, Chris Paul, Rob Sanson-Fisher

**Affiliations:** Priority Research Centre for Health Behaviour, Hunter Medical Research Institute, University of Newcastle, Callahan, Australia; Massachusetts General Hospital, United States of America

## Abstract

**Objective:**

To assess emergency physicians’ perceptions of individual and system enablers to the use of tissue Plasminogen Activator in acute stroke.

**Method:**

Australian fellows and trainees of Australasian College for Emergency Medicine completed a 57-item online survey assessing enablers to implementation of evidence-based practice across six domains: knowledge, skills, modelling, monitoring, feedback, and maintenance. Demographic and workplace characteristics were obtained. Descriptive statistics were calculated to describe demographic and workplace characteristics of responders, and survey responses. Each domain received an overall score (%) based on the number of responders agreeing with all items within the domain.

**Results:**

A total of 429 (13%) Australasian College for Emergency Medicine members responded. 17.7% of respondents reported they and/or their workplace met all knowledge-related enablers, however only 2.3% had all skill-related enablers in place. Of respondents who decide which patients receive tissue Plasminogen Activator treatment, 18.1% agreed that all maintenance-related enablers are in place at their hospital, compared to 6.6% for those who do not decide which patients receive tissue Plasminogen Activator treatment. None of the respondents had all items in place cross all domains.

**Conclusions:**

Even when allowing for the low response rate, it seems likely there is a lack of individual and system enablers supporting the implementation of best-practice stroke care in a number of Australian hospitals. Quality improvement programs could target all domains, particularly the skills-training and feedback emergency physicians receive, to aid implementation of tissue Plasminogen Activator treatment for acute stroke.

## Introduction

### Burden of the evidence-practice gap

Despite existence of evidence-based recommendations to guide clinical behaviour, these are often not adopted into practice [1]. It is estimated that 30–40% of patients do not receive potentially beneficial treatments [2]. Barriers and enablers to implementation of best-practice care have been identified at multiple levels, including the individual healthcare provider level, and the organisational and healthcare system level [3], [4]. Optimal implementation of best-evidence clinical practice requires changes across all stages [5].

### The evidence-practice gap in acute stroke treatment

Stroke is the third leading cause of disability-adjusted life years globally [6]. Use of thrombolytic therapy with intravenous tissue plasminogen activator (tPA) in appropriately selected acute ischaemic stroke patients is a powerful [7] and cost-effective [8] intervention. However, tPA carries a risk of brain haemorrhage [9]. In Australia, tPA is currently administered to 7% of ischaemic stroke patients [10], and 53% of hospitals offer this treatment [11]. Rates of tPA use are similar in other countries; in the United Kingdom 5% of stroke patients receive treatment [12], and 7% of ischaemic stroke patients are treated in the United States [13].

### Importance of the emergency department and emergency physicians in stroke care

Emergency department (ED) staff have an important role in the care of stroke patients. The urgent assessment and referral of stroke in ED in accordance with clinical practice guidelines is imperative for ischaemic stroke patients, particularly those eligible for tPA treatment. In hospitals without dedicated stroke specialists or stroke care units (SCU), emergency physicians may be responsible for the care and treatment provided to patients. Perceptions of the enablers of best-evidence clinical practice in acute stroke care have not yet been examined. The variance in uptake of tPA treatment highlights the need to explore individual and systems factors that affect the use of tPA in acute stroke care from the viewpoint of healthcare providers involved in the emergency care of patients.

### What individual-level enablers impact evidence-based practice?

Psychological theories of learning and behaviour including Social Learning Theory, Social Cognitive Theory, Operant Conditioning, and theory-driven frameworks such as The Behaviour Change Wheel, posit that a behaviour is more likely to be performed when an individual has the requisite knowledge and skills, and has seen the behaviour performed (modelled) [14]–[18].

Concrete and specific knowledge of what, when, by who, how a desired behaviour should be performed, and how often, is critical to the implementation of best-evidence practice [19] and can lead to improvements across quality indicators for emergency care [20]. Skills, including the opportunity to perform a desired behaviour, and to do so with cases of varying complexity, can increase confidence in performing behaviours [16]. In addition, modelling of behaviour by another can build both confidence and the necessary skills required to perform a behaviour [16]. Attitudes may also influence behaviour. For example, Grol found a higher rate of compliance with clinical guidelines when recommendations were supported by evidence, seen as non-controversial, compatible with current values, and beneficial to patients, compared to guidelines that were not based on research evidence, were seen as controversial, incompatible with current values, and potentially harmful to patients [14]. We have reported data on emergency physicians’ attitudes regarding the evidence, benefits and harms associated with tPA elsewhere, so these data are not reported in this manuscript.

### What system-level enablers may impact evidence-based practice?

Changes at the hospital or system-level may be necessary to facilitate the implementation of evidence-based practice [5]. Audit and performance monitoring have been shown to improve performance, provision of care, and patient outcomes [21]. A Cochrane Systematic Review provides evidence that the provision of regular feedback on health provider performance improves adherence to clinical practice guidelines [5], [22]. In addition, there is evidence to suggest that rewards at an individual and system level can improve the quality of healthcare [23].

System resources (for example, equipment, staffing, and additional support tools) are essential for the maintenance of evidence-based practice. Randomised controlled trials show that use of provider prompts, reminders, decision aids, and computer-assisted treatment plans are effective in improving adherence and maintenance to clinical practice guidelines [5], [24], [25]. The introduction of policies and protocols has also been shown to facilitate the implementation of recommended practice [5] and reduce adverse events [26].

The study successfully assessed emergency physicians’ perceptions of individual and system enablers in place to support the implementation of tPA in acute stroke care, including perceptions of the processes supporting knowledge, skills, modelling, monitoring, feedback and maintenance.

## Methods

### Setting

A web-based cross-sectional survey of emergency physicians and trainees in Australia was conducted.

### Ethics statement

The study was approved by Human Research Ethics Committee of the University of Newcastle and Australasian College for Emergency Medicine (ACEM) Scientific Committee.

### Participants

Australian fellows and trainees registered with ACEM were invited to take part. As all emergency physician fellows and trainees within Australia are registered with ACEM, this is an appropriate setting to assess perceptions of emergency physicians.

### Procedure

Fellows and trainees were sent an email from ACEM containing an information statement and link to the web-based survey. One reminder email was sent to all potential participants two weeks following initial contact. Completion of the survey was taken as implied consent.

### Measures

Participants completed a 57-item survey administered via Survey Monkey. Measures were derived from published literature on important features of behaviour change and implementation of evidence-based practice, in addition to recommended hospital facilities and evidence for tPA use according to National Stroke Foundation’s Clinical Guidelines for the management of Stroke [27]. Items were reviewed by two emergency physicians and two neurologists specialising in stroke at a tertiary hospital. The following data was collected:

#### Physician characteristics

Age, gender, role within the hospital, number of years worked in emergency care, role in stroke care, role in stroke treatment, and proportion of stroke patients they treat with tPA. De-identified data on the gender of all Australian fellows and trainees was obtained from ACEM to assess response bias. No other data were available for assessing bias.

#### Hospital characteristics

Whether arrangements for pre-hospital notification from ambulance are in place; number of ischaemic stroke patients presenting to ED per fortnight; proportion of stroke patients referred to an SCU or neurology department; presence of an SCU; presence of an intensive care unit; whether advanced imaging facilities are available; whether the hospital provides tPA treatment; proportion of ischaemic stroke patients receiving tPA treatment; proportion of emergency physicians giving tPA treatment; and whether the head of ED routinely gives tPA treatment.

#### Perceived enablers of tPA implementation

Forty items assessed participants’ perceptions of the degree to which enablers within six domains were in place within their workplace to support the implementation of tPA in stroke care. Respondents rated how much they agreed or disagreed with each item on a 5 point likert scale from strongly disagree [1] to strongly agree [5]. Domains included: Knowledge (9 items); Skills (6 items); Modelling (4 items); Monitoring (7 items); Feedback (8 items); and Maintenance (6 items).

### Data Analysis

For each item the responses ‘strongly agree’ or ‘agree’ were given a score of 1 and all remaining responses a score of 0. Given all domains are necessary according to behaviour change theories, each domain received an overall score (%) based on the number of responders agreeing with all items within the domain. Chi-square analyses were conducted to compare responses among participants that do and do not decide which patients receive tPA treatment, for individual items and overall domain scores.

## Results

Of the 3,280 invited ACEM members, 429 responded (response rate = 13%). Males (X^2^ (1, n = 3278) = 6.54, p = 0.01) were more likely to participate. Table 1 presents characteristics of responders.

**Table 1:** Demographic characteristics of respondents (n = 370).

Characteristic	Mean (SD)
Age	41.1 (8.2)
	**n (%)**
Male	256 (69.2)
Years worked in emergency care	
*≤5 years*	59 (15.9)
*5–10 years*	92 (24.9)
*11–15 years*	90 (24.3)
*≥16 years*	129 (34.9)
Role within the hospital (n = 359)	
*Emergency physician*	229 (63.8)
*Emergency physician trainee*	122 (34.0)
*Other*	8 (2.2)

Number of observations varies across characteristics due to missing data.

### Role of respondents in their workplace

81.6% of respondents reported they determine the care provided to stroke patients at their hospital and 32.4% indicated they hold responsibility for deciding which patients receive tPA treatment. Of those who decide which patients receive tPA, the median proportion of eligible patients treated with tPA was 15%.

### Workplace Characteristics

Table 2 presents respondents self-reported workplace characteristics. The median proportion of stroke patients referred to an SCU or neurology department from ED was estimated to be 85%. Of the hospitals that provide tPA treatment, the median proportion of ischaemic stroke patients treated with tPA was estimated to be 10%.

**Table 2:** Self-reported workplace characteristics of respondents (n = 359).

Characteristic	Mean (SD)
The average number of ischemic stroke patients seenby ED every fortnight	14.1 (12.9)
	**n (%)**
Arrangements are in place to receive pre-hospitalnotification of stroke patients from the ambulance service	238 (66.3)
The hospital has a dedicated stroke care unit (n = 358)	266 (74.3)
The hospital has an intensive care unit (n = 358)	338 (94.4)
The hospital has advanced imaging facilities (perfusionCT and MRI) (n = 358)	300 (83.8)
The hospital provides tPA treatment to eligible ischaemicstroke patients (n = 358)	278 (77.7)
The proportion of the emergency physicians at the hospitalwho routinely administer tPA treatment for eligible ischaemicstroke patients (n = 278)	
*None*	184 (66.2)
*Less than half*	33 (11.9)
*About half*	8 (2.9)
*Most*	36 (12.9)
*All*	17 (6.1)
The head of the emergency department routinely administerstPA treatment for eligible ischaemic stroke patients (n = 278)	
*Yes*	32 (11.5)
*No*	173 (62.2)
*I don’t know*	73 (26.3)

Number of observations varies across characteristics due to missing data and question applicability.

### Individual and system processes supporting the use of tPA

Table 3 shows responders’ agreement of the presence of potential enablers of tPA implementation. Results are shown separately for those who do and do not decide whether patients receive tPA, as well as for the total sample.

**Table 3:** Responders’ agreement of the presence of potential individual and system enablers of tPA implementation according to whether they are responsible for deciding which patients receive tPA treatment.

Statement	Decides tPA n (%) n = 116	Does not decide tPA n (%) n = 242	Total n (%)
**Knowledge**			[n = 429]
I can:			
Accurately identify stroke patients^*^	114 (98.3)	221 (91.3)	402 (93.7)
*Confidently interpret brain imaging scans*	78 (67.2)	161 (66.5)	287 (66.9)
Accurately identify which stroke patients may be eligible for tPA^*^	88 (75.9)	135 (55.8)	266 (62.0)
This hospital has a policy for:			
The management of stroke patients^*^	112 (96.6)	196 (90.0)	368 (85.8)
*Rapid referral of suspected stroke patients from ED to stroke specialists*	96 (82.8)	177 (73.1)	327 (76.2)
Rapid access to imaging for suspected stroke patients^*^	109 (94.0)	204 (84.3)	373 (87.0)
Administration of tPA when appropriate^*^	104 (89.7)	156 (64.5)	312 (72.7)
This hospital has:			
*Agreed indicators for quality of stroke care*	54 (46.6)	123 (50.8)	201 (46.9)
*Goals for improving performance on stroke care indicators*	48 (41.4)	93 (38.4)	168 (39.1)
**Skills**			[n = 415]
I have:			
*Undergone competency-based assessment for stroke protocol(s)*	10 (8.6)	16 (6.6)	29 (7.0)
*Undergone competency-based assessment for tPA use*	11 (9.5)	11 (4.6)	23 (5.5)
*Individual performance goals related to stroke care*	21 (18.1)	33 (13.6)	64 (15.5)
I regularly:			
Have the opportunity to develop my skills in stroke care^*^ ^†^	52 (44.8)	85 (35.1)	149 (37.9)
*Treat acute stroke patents* ^†^	109 (94.0)	219 (90.5)	359 (91.4)
*Have the opportunity to treat stroke cases of varying complexity* ^†^	107 (92.4)	211 (87.2)	347 (88.3)
**Model**			[n = 415]
Respected and influential members of this hospital:			
Endorse the use of tPA^*^	94 (81.0)	146 (60.3)	277 (67.5)
*Actively model best practice stroke care to all staff*	61 (52.6)	103 (42.6)	180 (43.9)
I have:			
*Seen tPA administered to stroke patients on several occasions*	94 (81.0)	185 (76.5)	327 (78.8)
Received interactive training in tPA administration^*^	35 (30.2)	39 (16.1)	86 (20.8)
**Monitoring**			[n = 393]
Respected and influential members of this hospital monitor:			
*My performance of stroke care*	29 (25.0)	43 (17.8)	81 (20.6)
*The hospitals’ performance on key stroke care indicators*	46 (39.7)	87 (36.0)	147 (37.4)
*Actions which are inconsistent with guideline care for stroke patients*	36 (31.0)	65 (26.9)	111 (28.3)
The proportion of eligible stroke patients who receive tPA^*^	45 (38.8)	76 (31.4)	136 (34.6)
This hospital has a system in place for:			
*Implementing action plans for improving performance for stroke care*	48 (41.4)	83 (34.3)	144 (36.6)
*Reviewing outcomes of quality improvement plans for stroke care*	44 (37.9)	72 (29.8)	127 (32.3)
Entering data from all patients treated with tPA into a central register^*^	37 (31.9)	49 (20.25)	97 (24.7)
**Feedback**			[n = 375]
I am regularly given:			
*Individual performance feedback following relevant cases of acute stroke* ^†^	10 (8.6)	16 (6.6)	32 (8.1)
This hospital regularly receives feedback on:			
*Its performance on stroke care indicators*	17 (14.7)	35 (14.5)	53 (14.1)
*Its performance on stroke care indicators compared to other hospitals*	13 (11.2)	32 (13.2)	46 (12.2)
Excellence in stroke care performance is rewarded at:			
*The hospital level*	19 (16.4)	28 (11.6)	48 (12.8)
*An individual level*	11 (9.5)	12 (5.0)	25 (6.6)
If I do not follow tPA protocol, there are negative consequences for:			
*Me*	35 (30.2)	78 (32.2)	118 (31.5)
*The hospital*	33 (28.5)	49 (20.3)	84 (22.4)
*The patient*	53 (45.7)	86 (35.5)	143 (38.1)
**Maintenance**			[n = 375]
To help me follow stroke care protocol there are:			
Checklists/decision aids to help identify and triage a possible stroke case^*^	92 (79.3)	151(62.4)	258 (68.8)
Checklists/decision aids to help identify stroke patients eligible for tPA^*^	95 (81.9)	139(57.4)	250 (66.7)
Checklists/decision aids to help interpret imaging for a possible stroke case^*^	34 (29.3)	38 (15.7)	78 (20.8)
At all times I have immediate access to:			
*Advice from a senior colleague in managing stroke^#^*	95 (81.9)	179 (74.0)	286 (76.9)
*Brain imaging facilities and staff trained to interpret images^#^*	99 (85.3)	181 (74.8)	292 (78.5)
*A multidisciplinary acute care team trained in the delivery and monitoring of tPA^#^*	54 (46.6)	97 (40.1)	156 (42.0)

Number of observations varies across items due to missing data. ^*^p<0.05; ^†^n = 393 total; ^#^n = 372 total.

### Domain scores

**Figure 1:**
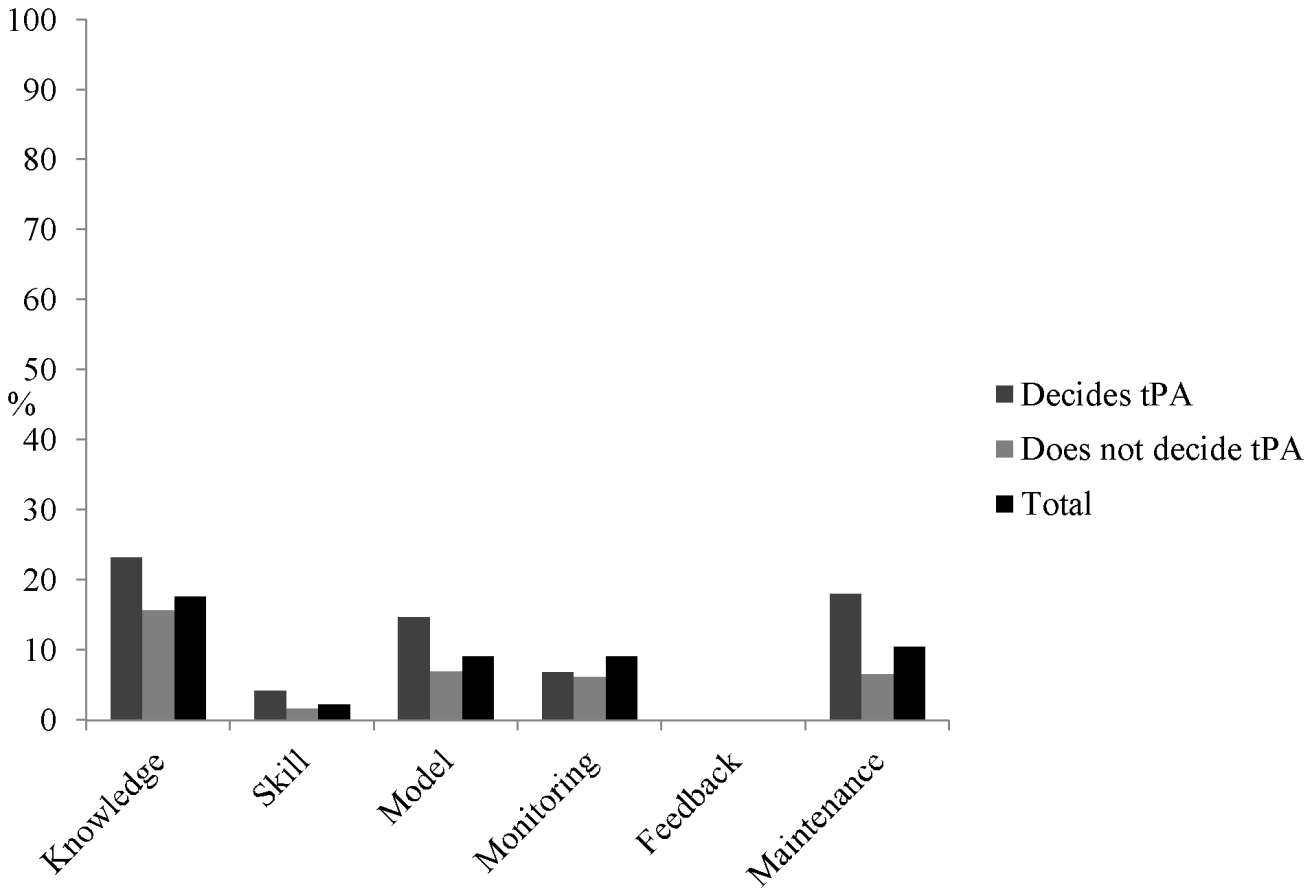
Percentage of respondents who agree all enablers within a domain are present for each domain, according to whether they are responsible for deciding which patients receive tPA treatment.

Fig. 1 outlines the overall score for each domain based on respondents’ agreement that all enablers are in place within their hospital. Results are shown separately for those who do and do not decide whether patients receive tPA, as well as for the total sample. For the total sample, the proportion of responders indicating each enabler was present within each domain ranged 0–17.7%, with no responders indicating that all enablers are in place across all of the domains. When comparing domain-ratings between those that do and do not decide which patients receive tPA treatment, significant differences were found for the proportion of responders reporting the presence of model (X^2^ (1, n = 358) = 5.31, p = 0.02) and maintenance enablers (X^2^ (1, n = 358) = 11.17, p = 0.00).

## Discussion

To our knowledge, this is the first study to assess perceptions of individual and system enablers in place to support the implementation of tPA in acute stroke care among Australian emergency physicians. We encountered a low response rate (13%), limiting the generalizability of our results. Examination of the responding physician and workplace characteristics indicates hospitals that already administer tPA treatment to ischaemic stroke patients may be over-represented here. Therefore, it is likely the majority of results apply to this sub-group. Even when allowing for the low response rate, results of the study suggest few of the recommended individual and system enablers supporting the implementation and maintenance of best-practice stroke care are consistently in place in Australian hospitals.

### Physician characteristics

While the majority of respondents (81.6%) reported being involved in determining the care provided to stroke patients, less than half (32.4%) reported they were responsible for deciding which patients receive tPA treatment. This difference suggests the decision to treat may fall on only a small portion of emergency physicians, and may be due to hospital referral procedures, as well as the use of telehealth for specialist consultations.

### Workplace characteristics

About three quarters (77.7%) of the responders indicated their hospital provides tPA treatment to eligible stroke patients. This proportion is higher than the 53% reported by National Stroke Foundation [11]. While tPA is becoming increasing available, this difference may represent a response bias. That is, individuals working in hospitals providing tPA may have been more likely to respond than those working in hospitals that do not provide tPA.

### Health provider care (Individual factors)

#### Knowledge

Only 62.0% and 66.9% of responders reported sufficient knowledge to identify stroke patients eligible for tPA treatment, and interpret brain imaging scans, respectively. Of those that reported they are responsible for deciding which patients receive tPA, 75.9% reported sufficient knowledge to identify patients eligible for tPA, compared to 55.8% of those not responsible for deciding treatment. Significant differences in responses among those who do and do not decide which patients receive tPA were found for this enabler. While it is acknowledged that not all responders are responsible for the decision to treat a patient with tPA, these deficits in knowledge could contribute to the evidence-practice gap, given the time urgency and team nature of the care provided to stroke patients. Deficits in this enabler are highly modifiable as Continuing Medical Education (CME) can increase health care provider knowledge, attitudes and skills, also resulting in improved patient outcomes [28].

#### Skills

Results suggest limited support was in place for building upon healthcare providers’ skills in relation to tPA. Few respondents (5.5%−7.0%) agreed with the statements “I have undergone competency-based assessment for tPA use” and “I have undergone competency-based assessment for stroke protocol(s)”. The absence of competency-testing and skill development is concerning given the ability to perform a task is believed a necessary pre-requisite for behaviour change [16]. Particular to the treatment of stroke, a ‘learning culture’ is significantly associated with the likelihood of receiving thrombolysis [29]. Learning approaches that incorporate multi-media, occur on multiple occasions, and are interactive, produce greater behaviour change in relation to clinical performance [28].

#### Modelling

With the exception of “Respected and influential members of this hospital actively model best practice stroke care to all staff” and “I have received interactive training in tPA administration”, there was relatively high agreement (67.5–78.8%) with items assessing the presence of modelling. However, of those who are responsible for deciding which patients receive tPA, 81% reported respected and influential members of their hospital endorse the use of tPA compared to only 60.3% for those who are not responsible for deciding which patients receive tPA. Significant differences in responses among those who do and do not decide which patients receive tPA were found for this enabler. This is important as the presence of individual clinical leadership in a hospital setting is associated with the likelihood of receiving tPA treatment [29]. Modelling is particularly effective when undertaken by respected and influential leaders [30].

### Hospital system processes (Workplace factors)

#### Monitoring

An absence of performance monitoring in relation to stroke care within the workplace was reported by respondents as only 20.6%–37.4% agreed with items addressing this domain. This included monitoring of: their hospitals’ performance, the rates of tPA administration, and actions deemed inconsistent with guideline care.

#### Feedback

Few respondents (8.1%–14.1%) indicated that feedback on stroke care performance at the individual and hospital level was provided by their workplace. Previous research has highlighted the importance of constructive feedback for implementing guideline practice [16] as a recent Dutch cohort study of hospitals admitting acute stroke patients found a significant association between thrombolysis rates and the availability of informal and formal feedback [29].

#### Maintenance

Provider focussed decision aids increase adherence to guidelines [25] by prompting healthcare providers to follow clinical practice guidelines. Our results indicate, however, that while most physicians (66.7%) have checklists/decision aids to help identify stroke patients eligible for tPA, the presence of this enabler significantly varies amongst those who are (81.9%) and are not responsible (57.4%) for deciding which patients receive tPA. In addition, the majority of respondents (56.6%) do not have access to checklists/decision aids to assist with interpretation of brain imaging. The organisation of health systems, particularly introduction of a stroke recognition tool [31], in addition to an Acute Stroke Team emergency call system [32], produced an increase in the diagnostic accuracy of stroke among ED staff [31] and a reduction in time from patient arrival at hospital to CT scan [32]. This in turn has been shown to increase rapid and appropriate referral of stroke patients to specialists, resulting in timely treatment, and better outcomes for patients [31]–[33].

### Behaviour Change Domains

When comparing domain-ratings between those that do and do not decide which patients receive tPA treatment, significant differences were found for the presence of model and maintenance enablers. This may reflect that those who are responsible for making decisions about tPA have greater awareness of enablers within their workplace, or alternatively that workplaces which have such enablers in place are more likely to support their staff to make decisions about tPA.

The latter interpretation is consistent with evidence from prior studies regarding the role of system enablers in supporting evidence-based care. Optimal implementation and maintenance of best-evidence clinical practice requires changes within both the healthcare provider and the system [5]. As such, the reason for low rates of tPA use across Australia could be that all enablers of evidence-based practice are not in place in hospitals. The sum of the cultural characteristics facilitating the use of tPA treatment within a hospital is associated with the rate of thrombolysis administration [29]. This suggests, the more individual and system enablers in place to support the use of tPA, the more likely a patient is to receive care in accordance with clinical practice guidelines.

### Limitations

The study yielded a low response rate (13%), similar to other online surveys of physicians [34], and therefore results are limited in their generalisability. Given the likelihood that hospitals already administering tPA treatment to ischaemic stroke patients are over-represented in the sample, the true proportion of enabling factors in Australian hospitals may be lower than that reported. The self-reported nature of workplace characteristics must also be taken into consideration when interpreting results. Finally, New Zealand members of ACEM were sent an invitation email. While it is possible results contain data from a number of New Zealand participants (estimated less than 1.5% of responders), the invitation email was rescinded immediately. This is highly unlikely to have had a significant impact on survey results.

### Conclusions and Implications

This is the first study to explore individual factors and system enablers in place to support use of tPA in acute stroke care from the perception of Australian emergency physicians. Our results are supported by previous international literature relating to workplace resources for stroke care, physician perceptions of tPA use, and strategies for producing behaviour change. While enablers mapping to the domains of skill and monitoring are somewhat present according to emergency physicians, there appears to be a lack of feedback and maintenance enablers in place in hospitals across Australia for the implementation of this evidence-based treatment. In addition the reported presence of model and maintenance-related enablers varies amongst responders who do and do not decide which patients receive tPA treatment.

As emergency physicians are commonly the first point of contact for the in-hospital care of stroke patients, their perceptions towards tPA use, and the system processes to support its use, in acute stroke may be a critical factor in the adoption and guideline use of this treatment. Results suggest that quality improvement programs could use specific strategies that target emergency physicians’ knowledge and skills, modelling and monitoring of the behaviour, as well as provision of feedback, and maintenance systems to aid the implementation of tPA treatment for acute stroke.
